# A Sensitivity-Enhanced Film Bulk Acoustic Resonator Gas Sensor with an Oscillator Circuit and Its Detection Application

**DOI:** 10.3390/mi8010025

**Published:** 2017-01-19

**Authors:** Mengying Zhang, Lidong Du, Zhen Fang, Zhan Zhao

**Affiliations:** 1 State Key Laboratory of Transducer Technology, Institute of Electronics, Chinese Academy of Sciences, Beijing 100190, China; zhangmengying12b@mails.ucas.ac.cn (M.Z.); lddu@mail.ie.ac.cn (L.D.); zfang@mail.ie.ac.cn (Z.F.); 2 University of Chinese Academy of Sciences, Beijing 100080, China

**Keywords:** film bulk acoustic resonator (FBAR), adsorption, humidity sensor, oscillator circuit

## Abstract

This paper presents a sensitivity-enhanced gas sensor based on a film bulk acoustic resonator (FBAR). It was designed and fabricated with micro through-holes in its top electrode for sensitivity enhancement. The sensor was driven by a Colpitts oscillator circuit, and the output signal had characteristics of a power of −2.6 dBm@3 V and a phase noise of −90 dBc/Hz@100 kHz. In order to test the performance of the sensor, it was used for the detection of relative humidity (RH) and ethanol. When the relative humidity ranged from 25% to 88%, the frequency shift of the sensor was 733 kHz, which was 3.2 times higher than that of the existing FBAR sensor with a complete top electrode. Fitting results of the frequency shift and the relative humidity indicated that the measurement error was within ±0.8% RH. When the ethanol concentration ranged from 0 to 0.2355 g/L, the frequency shift of the sensor was 365 kHz. The effect of the oscillator circuit on the adsorption reaction and temperature response of the FBAR sensor device was analyzed to optimize its detection application.

## 1. Introduction

Sensors based on a film bulk acoustic resonator (FBAR) have attracted plenty of attention because of their highly sensitive resonance frequency to various parameters, such as mass [1], pressure [2], temperature [3] and light [4,5]. Compared with capacitive sensors and resistance sensors, the frequency signal of FBAR sensors is detected with high accuracy and precision. The quartz crystal microbalance (QCM) is another bulk acoustic resonator, and it has been widely used as a mass sensor. The structures and the working principles of FBAR and QCM are similar. Decided by the piezoelectric layers, the resonance frequency of the QCM is usually from 5 to 10 MHz, while that of the FBAR reaches GHz. With a similar adsorption reaction, the mass sensitivity and discernibility of FBAR sensors are much higher than those of QCM sensors. Take humidity detection, for example, with ZnO nanostructures or some organic materials as sensitive layers [6,7,8,9,10]; the sensitivity of the QCM sensor was enhanced to −77 Hz/% RH [11]. Meanwhile, the sensitivity of the FBAR humidity sensor reached −43 kHz/% RH with a resonance frequency of 6 GHz [12]. In addition, the micro-electro-mechanical systems (MEMS) process for FBAR is compatible with the semiconductor process, so FBAR sensors and the circuit can be easily integrated in a single chip [13] to obtain the lab-on-a-chip. So FBAR sensors have potential for excellent performance and wide application. Research on them has focused on the characteristics of their sensitive response and the detection of this response. 

When a FBAR sensor is used in gas detection, its response to the gas depends on the mass sensitivity of the resonator and the adsorption reaction on its sensitive surface [14]. Compared with the former one, the relationship between the concentration of the gas and the mass of the adsorbed phase is more complex, and it depends on the characteristics of the adsorbent [15], the state of the sensitive surface and the environmental parameters [16]. Many works on resonator gas sensors have focused on their adsorption reaction for sensitivity enhancement. The common method was making an additional highly sensitive layer, which was porous [17], nanostructured [18] or had a good chemical reaction with the adsorbate [19], on the surface of the sensor. In the literature, the response of the resonator gas sensors to different gases was presented [20,21,22,23], but the exact relationship between the frequency shift and the concentration of the gas, which was necessary for the detection application, was seldom defined. 

The frequency of FBAR sensors requires great effort to be detected, especially when it is above 1 GHz. With an advanced instrument, such as a network analyzer, it was detected in the laboratory [24,25]. To expend their detection application, an oscillator circuit for the FBAR sensors is important. Exploration of the resonator’s lumped element equivalent model had been reported for the circuit design [26,27]. An oscillator circuit with FBARs had been fabricated with different structures [13,28,29]. Furthermore, the performance of the integrated sensor will be affected by its circuit section. It had been reported that the supplied voltage of the oscillator circuit changed its frequency and this effect was used for the temperature compensation of the FBAR sensor [30]. However, there have been few studies about the effect of the circuit on the response of the FBAR sensor. 

In this work, a sensitivity-enhanced FBAR gas sensor was designed and fabricated. It was driven by a Colpitts oscillator to obtain a frequency signal for detection application. Then the FBAR sensor was used to detect relative humidity and ethanol. The effect of the oscillator circuit on the response of the sensor was analyzed to optimize its detection application.

## 2. Materials and Methods 

### 2.1. The FBAR Sensor Chip

**Figure 1:**
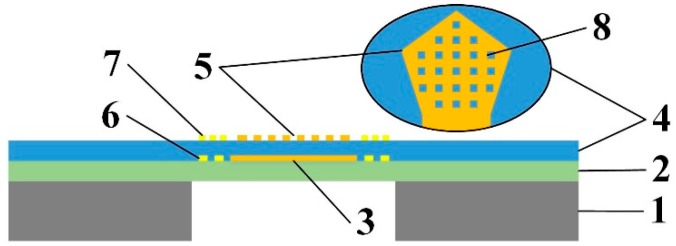
Cross-section of the film bulk acoustic resonator (FBAR) chip with detail of the micro through-holes in top electrode. 1—Silicon substrate; 2—Silicon nitride support film; 3—Bottom electrode; 4—ZnO piezoelectric film; 5—Top electrode; 6—Resistor heater; 7—Resistor temperature sensor; 8—Micro through hole.

The FBAR sensor chip designed for gas detection was shown in Figure 1. The FBAR multilayer-film structure, consisting of two electrodes and a piezoelectric film between them, was made on a silicon nitride support film and a silicon substrate. Center of the substrate was removed to release a suspending area for the resonator. A micro resistor temperature sensor and a micro resistor heater were placed around the resonator in the suspending area for temperature monitoring and control. 

Performance of the FBAR sensor is decided by the material and the structure. ZnO is semiconductor material with piezoelectric properties [31]. For wurtzite phase ZnO, the piezoelectric constant of the *c*-axis, namely (002) orientation, is the largest among all the crystal orientation. So *c*-axis-oriented polycrystalline ZnO film was chosen as the piezoelectric layer in the presented FBAR sensor for best piezoelectric properties. Furthermore, there are sensitive chemical adsorption and strong physical adsorption between crystalline ZnO and some kinds of molecules, such as water [32,33], ozone [34], hydrogen [35] and ethanol [36]. ZnO film in the sensor acted as both piezoelectric layer and sensitive layer. 

**Figure 2:**
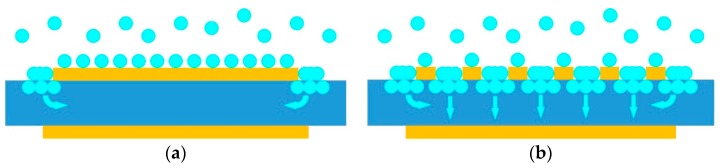
Schematic diagrams of adsorption on surface of the FBAR sensors: (**a**) model with complete electrode; (**b**) model with micro through-holes in top electrode.

For sensitivity enhancement, micro through holes with size of 10 μm × 10 μm were made in the top electrode. As shown in Figure 2, when the top electrode is complete, most of the molecules adsorb on surface of the electrode and only a few ones adsorb on the ZnO crystal at the edges. With micro through holes in the top electrode, more and stronger adsorption occurs on the exposed ZnO surface, and the molecules diffuse into the polycrystalline film through grain boundaries. By comparison, the presented FBAR sensor with micro through-holes in top electrode obtains more mass loading and higher sensitivity than the existing one.

**Figure 3:**
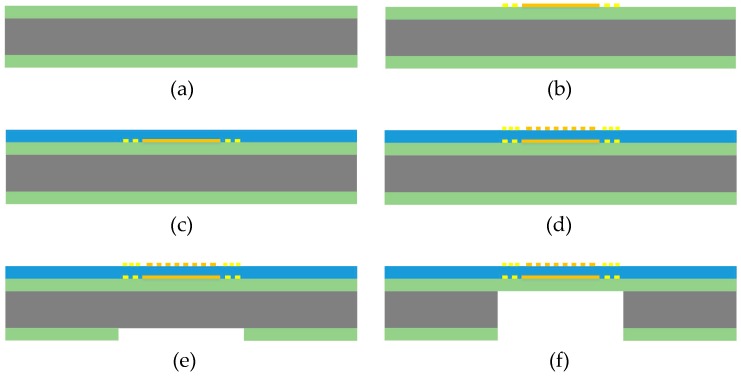
Fabrication process for the sensor chip.

The newly designed FBAR sensor chip was fabricated with the MEMS process in Figure 3.

(a) First, 1.5 μm silicon nitride film was deposited on the silicon substrate by low pressure chemical vapor deposition (LPCVD).
(b) Pt film for bottom electrode and resistor heater was deposited on top surface of the silicon nitride film by physical vapor deposition (PVD) and patterned.
(c) Then 1.2 μm ZnO film was sputtered on the top surface of the chip and patterned.
(d) Pt film for top electrode and resistor temperature sensor was deposited on surface of the ZnO film and patterned.
(e) On the back of the chip, silicon nitride film in suspending area was etched by reactive ion etching (RIE).
(f) With the patterned silicon nitride film as mask, silicon was etched from the back by deep reactive ion etch (DRIE), until it reached the top silicon nitride film.

**Figure 4:**
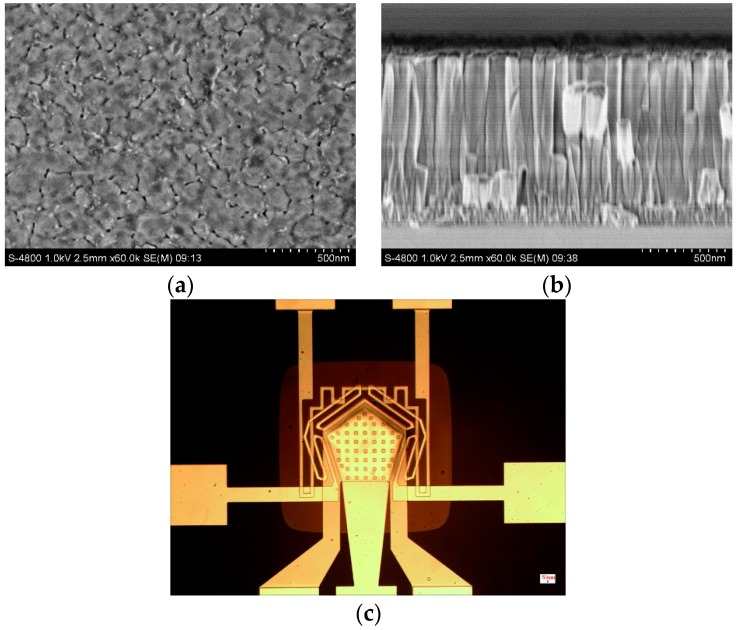
Scanning electron microscope (SEM) photos of the ZnO film: (**a**) top view; (**b**) cross-section; (**c**) the microscopic photo of the fabricated FBAR chip.

As shown in Figure 4a,b, the ZnO film deposited on this chip obtained porous surface and highly *c*-axis-oriented polycrystalline structure. So it had strong adsorption as a sensitive layer and good piezoelectric properties as a piezoelectric layer. The fabricated chip was shown in Figure 4c. The films were smooth and the edges of the patterns were clear, especially of the through holes.

### 2.2. The Oscillator Circuit

**Figure 5:**
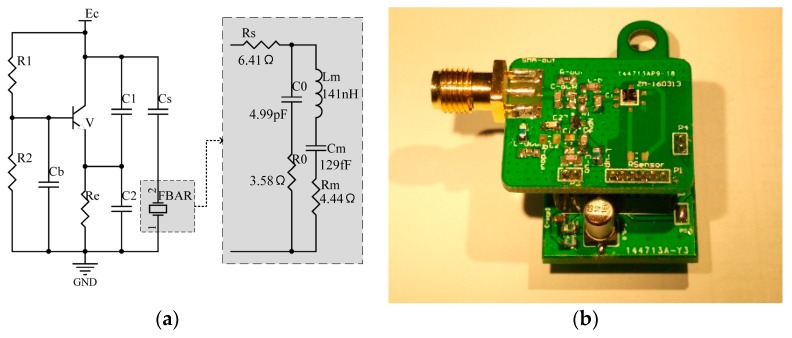
(**a**) Schematic diagram of the oscillator circuit and (**b**) the photo of a sensor sample.

To detect frequency of the FBAR sensor, it was connected into a Colpitts oscillator circuit, as Figure 5a shows. In the block, the FBAR was represented by the Modified Butterworth Van-Dyke (MBVD) model and parameters in the model were obtained by measuring impedance characteristics of the fabricated FBAR chip. Then the circuit acted as a Seiler oscillator and its frequency was approximately the one of the FBAR sensor. Printed circuit boards (PCBs) of the oscillator circuit were fabricated. A FBAR chip was attached to the PCB and pads of them were connected by wire bonding. In Figure 5b, the sample consisting of the FBAR sensor and the oscillator circuit was used for gas detection.

### 2.3. Methods

Impedance characteristics of the FBAR were measured by a Network Analyzer (Agilent Technologies, Santa Clara, CA, USA, E5061B). Signals output from the sensor samples were detected with a CXA signal analyzer (Agilent Technologies, N9000A) and a Mixed Signal Oscilloscope (Keysight Technologies Inc., Santa Rosa, CA, USA, ms071604c). In relative humidity detection, humidity and temperature were controlled by a Humidity Generator. Detections of the sensor in this work were carried out in static state.

## 3. Results and Discussion

### 3.1. Frequency Signal of the Sensor Sample

**Figure 6:**
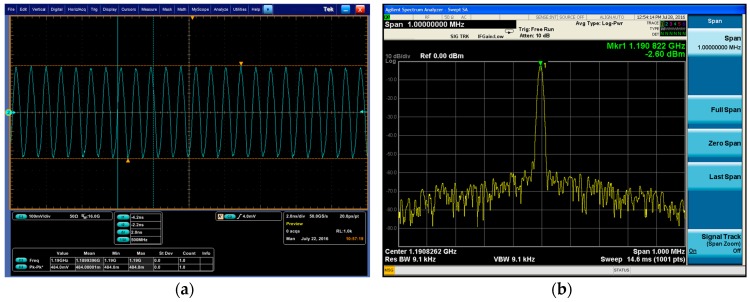
Detection of the signal output from the sensor sample: (**a**) oscillogram; (**b**) spectrum.

The signal output from the sensor sample was detected and the results are shown in Figure 6. With a supplied voltage of 3 V, the peak-to-peak value of the waveform was 484 mV. In the spectrum, the power of the signal was −2.6 dBm and the phase noise was −90 dBc/Hz@100 kHz. The frequency signal was strong with a high-quality detection application of the sensor. 

### 3.2. Response to Humidity

**Figure 7:**
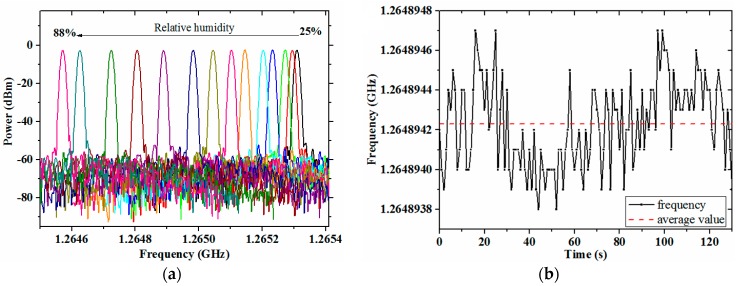

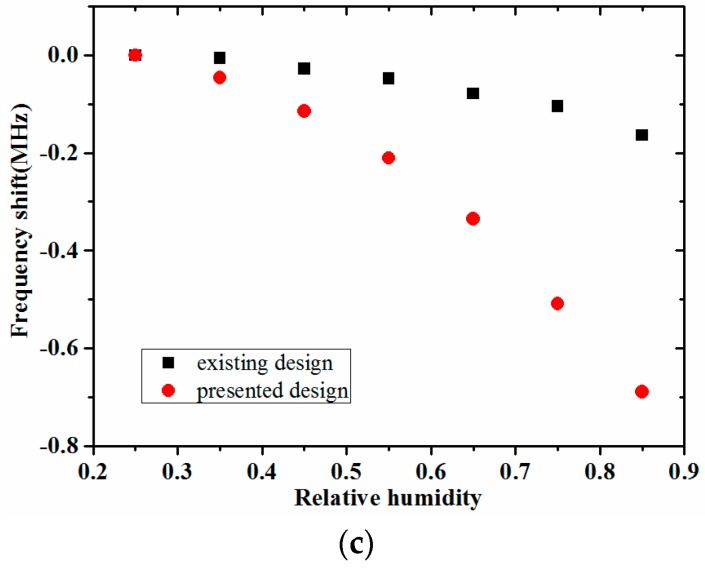
Relative humidity detection results of the FBAR sensor samples: (**a**) resonant peak shifts of the presented sensor and (**b**) random variation of its frequency; (**c**) comparison of the frequency shifts.

The presented FBAR sensor sample was used for the detection of the relative humidity. As shown in Figure 7, when the relative humidity ranged from 25% to 88% at 25 °C, the frequency shift was 733 kHz. Random variation of the sample’s frequency with stable humidity and temperature was within 1 kHz, which meant a high resolution of the sensor. For comparison, the sample with the existing FBAR sensor chip was fabricated and detected, and its frequency shift was 164 kHz. It indicated that the presented sensor obtained a higher sensitivity than the existing one.

When FBAR sensors are applied to mass measurement based on the adsorption reaction, their response to the substance is composed of two parts: the response of the resonance frequency to the mass loading, and the relationship between the adsorption mass and the concentration of the adsorbate. 

For the former, the relationship between the frequency shift and mass loading is linear, according to the Sauerbrey equation as shown in Equations (1)–(3). In these equations, *v*, *t*, *N*, ρ and *S* stand for the acoustic velocity, thickness, frequency constant, density, and acreage of the resonator, respectively. For a small relative mass change, m≫∆m, the frequency shift proportional to the change of mass was obtained.

(1) $$f=\frac{v}{2t}=\frac{N}{t}=\frac{N\rho S}{m}$$

(2) $$∆f=N\rho S\left(\frac{1}{m+∆m}-\frac{1}{m}\right)=-\frac{{f_{0}}^{2}}{N\rho S}\frac{∆m}{1+\frac{∆m}{m}}$$

(3) $$∆f\approx -\frac{{f_{0}}^{2}}{N\rho S}∆m$$

For the latter, the relationship between the mass of the adsorbed phase and the partial pressure of the adsorbate is nonlinear, according to the Freundlich equation. In Equation (4), Г is the adsorption mass with the adsorbent of unit mass; *P* is the partial pressure of the adsorbate and *P*_0_ is the saturated vapor pressure at the ambient temperature; *k* and *n* are constants of the adsorption reaction. For parameter *n*, a smaller one means easier adsorption.

(4) $$\Gamma =k{\left(\frac{P}{P_{0}}\right)}^{n}$$

Combining the two parts, the relationship between the frequency shift and the concentration of the adsorbate is obtained as Equation (5). 

(5) $$∆f=-\frac{f^{2}k}{N\rho S}{\left(\frac{P}{P_{0}}\right)}^{n}$$

**Figure 8:**
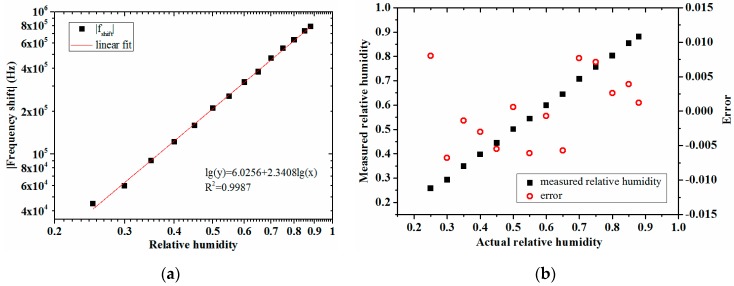
Analysis of the presented sensor’s response to relative humidity: (**a**) linear fit between lg(*f*_shift_) and lg(RH); (**b**) the measurement error.

According to this relationship, the linear fit between lg(*f*_shift_) and lg(RH) was done with the measurement results of the presented sensor, as shown in Figure 8a. The response of the sensor sample to the relative humidity is quantified in Equation (6). Comparing the measurement humidity calculated by the equation with the actual humidity, the measurement error was ±0.8% RH, as Figure 8b shows.

(6) $$f_{\mathrm{shift}}=-1060718\times {\left(\mathrm{RH}\right)}^{2.3408}$$

The detection results showed that the presented FBAR sensor has high accuracy, sensitivity, and resolution.

### 3.3. Response to Ethanol

**Figure 9:**
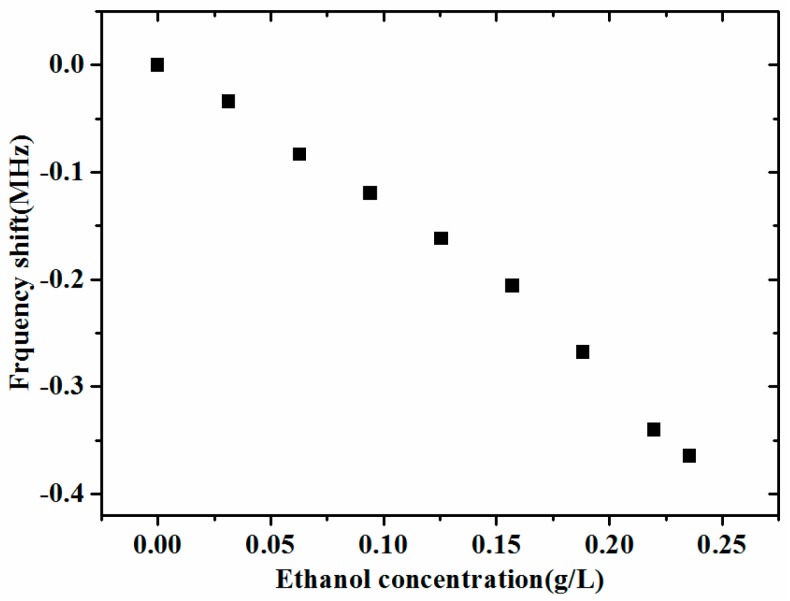
The frequency shift of the presented sensor sample with changed ethanol concentration.

The sensor sample was used for ethanol detection at room temperature. Its frequency shift was 365 kHz with the ethanol concentration ranging from 0 to 0.2355 g/L, as Figure 9 shows. The phenomenon of saturation appeared at the concentration of 0.2355 g/L. It indicates that, with the sensitivity-enhancement design, the presented FBAR sensor has the capability to detect gases which are able to react with ZnO.

### 3.4. Oscillator Circuit Effect on the Response

#### 3.4.1. Sensitivity of the Sensor

The response of the FBAR gas sensor is based on adsorption on its surface. The adsorption will be affected by the state of the surface. Yeh et al. [37] reported that the vibration of the piezoelectric film assisted the desorption of the nanomolecules with the effects of the electric field and acoustic streaming. When a FBAR sensor works at the resonance frequency, molecules adsorbed on its surface get additional energy. More molecules reach the activation energy of desorption and the reversible adsorption reaction tends toward desorption. The energy of the molecules is increased by the resonance of the piezoelectric film by two mechanisms. One is the effect of the mechanical movement. In the vibration, the surface of the resonator moves periodically and molecules adsorbed on it obtain kinetic energy. In addition, part of the energy translates into internal energy by friction from the viscosity. The other mechanism is the effect of the alternating electric field. When the piezoelectric film resonates, alternating charges and an electric field generate on the surface. Polar molecules, such as water, on or near the surface oscillate with the electric field and their energy increases due to friction between them. For the two aspects, a high frequency and large amplitude of the resonance are both beneficial for the increase of the energy and the desorption of the molecules. As a result, the mass of the adsorbed phase decreases, as does the sensitivity of the sensor. 

**Figure 10:**
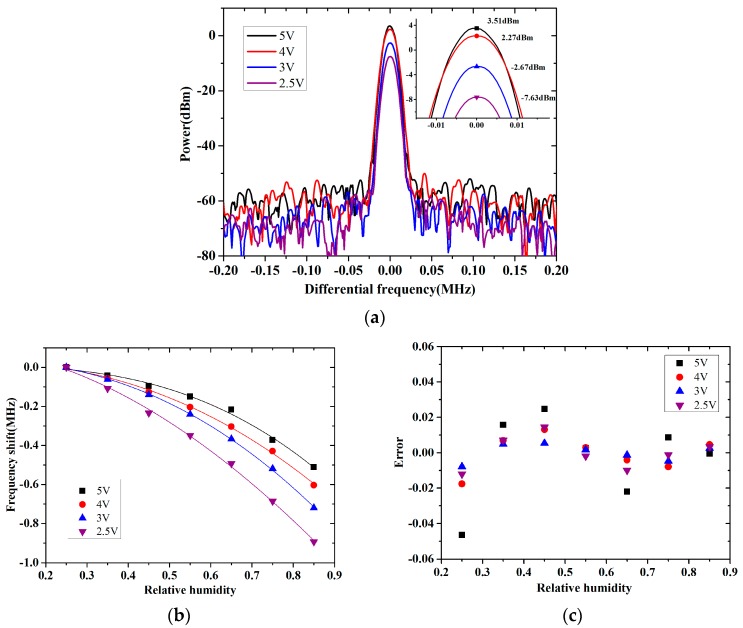
Effect of the supplied voltage on output frequency signal of the sensor sample: (**a**) power of the signal; (**b**) the frequency shift with relative humidity; (**c**) measurement error.

When the sensor sample was used for humidity detection, a different voltage was supplied to the oscillator circuit. Except for the frequency, the power of the output signal changed, as shown in Figure 10a. It meant that the oscillation amplitude of the resonator varied with the supplied voltage of its oscillator circuit. The responses to the relative humidity of the sensor sample with different supplied voltages were detected. As shown in Figure 10b, when the supplied voltage decreased from 5 to 2.5 V, the frequency shift for the detected range increased and the parameter *n* in Equation (5) decreased from 2.7 to 1.8, which meant that adsorption on surface of the sensor became easier. The measurement results matched the analysis above. Of all the measurement errors in Figure 10c, the one with the 3 V supplied voltage had the smallest error value. It indicated that a low supplied voltage of the circuit was beneficial for the high sensitivity of the sensor, but the accuracy could be reduced when it was too low. The analysis was useful in choosing the supplied power for the sensor to optimize its detection application. 

#### 3.4.2. Response to Temperature

**Figure 11:**
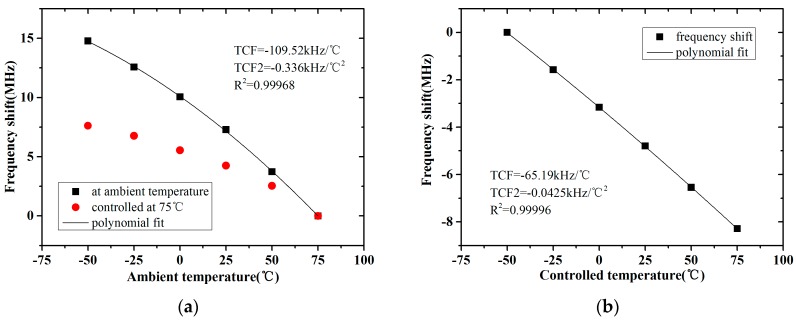
The response of the sensor sample to temperature: (**a**) one with different ambient temperatures; (**b**) one with resonator controlled at different temperatures.

When the temperature changes, except for the resonator, the components and parasitic parameters in the oscillator circuit are affected. Then response of the sensor sample to temperature is divided into the two sections. As shown in Figure 11a, at different ambient temperatures, the frequency shifts of a sensor sample and the same sample with the resonator controlled at 75 °C by the integrated thermal resistors were detected. Between them, the former one was the temperature response of the whole sample and the latter one was the response of the circuit section. To obtain the temperature response of the resonator section, the sensor sample was placed at −50 °C, and the temperature of its resonator was controlled at different values, as Figure 11b shows.

Kropelnicki et al. [38] pointed out that the frequency shift of a FBAR sensor with temperature could be approximated by a second-order Taylor polynomial. For predigestion, the nonlinear response of the whole sample and the FBAR in it to temperature were both approximated by a second-order Taylor polynomial with a first-order temperature coefficient of frequency (TCF) and a second-order temperature coefficient of frequency (TCF2), as shown in the diagrams. 

In this study, the temperature response of the circuit section and the resonator section in the sensor sample were distinguished and quantified. It was useful to analyze each of them to obtain and optimize the temperature performance of the sensor. 

## 4. Conclusions

A sensitivity-enhanced gas sensor based on a FBAR was designed and fabricated in this work. Micro through-holes were made in its top electrode to enhance adsorption on the surface. Sensor samples consisting of the FBAR sensor and the oscillator circuit were fabricated and detected. The frequency signal output from the sample was strong with a high-quality detection application of the sensor. As a gas sensor based on adsorption, the response of the FBAR sensor to gases was divided into two parts to analyze the relationship. When the sensor samples were used for RH detection, the frequency shift of the presented FBAR sensor was 733 kHz with a relative humidity from 25% to 88%, which was 3.2 times larger than that of the existing FBAR sensor with a complete electrode. The minimum detectable frequency shift of the output signal was 1 kHz. The relationship between its frequency shift and relative humidity was fit according to the Freundlich equation. Its measurement error was within ±0.8% RH. The results indicated that the presented FBAR sensor had high accuracy, sensitivity, and resolution. In ethanol detection, the presented sensor obtained a good response with a 365 kHz frequency shift for the detected range. With the sensitive enhancement design, the presented FBAR sensor had the potential to gases able to react with ZnO. The effect of the oscillator circuit on the sensitivity and temperature response of the FBAR sensor device was analyzed. This study proposed the method of adjusting the oscillator circuit of the FBAR sensor to optimize its detection application.
